# Menstrual hygiene practices among adolescent women in rural India: a cross-sectional study

**DOI:** 10.1186/s12889-022-14622-7

**Published:** 2022-11-19

**Authors:** Aditya Singh, Mahashweta Chakrabarty, Shivani Singh, Rakesh Chandra, Sourav Chowdhury, Anshika Singh

**Affiliations:** 1 Banaras Hindu University, Varanasi, Uttar Pradesh India; 2 India Health Action Trust, Lucknow, Uttar Pradesh India; 3 Tata Institute of Social Science, Mumbai, Maharashtra India; 4 Raiganj University, Raiganj, West Bengal India

**Keywords:** Menstrual hygiene, Hygienic methods, Sanitary napkins Adolescent women, Rural India, India

## Abstract

**Background:**

Exclusive use of hygienic methods (sanitary napkins, locally prepared napkins, tampons, and menstrual cups) to prevent the visibility of bloodstains during menstruation is still considerably low among adolescent women in rural India. However, no prior research has explored the prevalence and determinants of exclusive hygienic methods among rural Indian adolescent women. To address this gap, this study examines the factors affecting adolescent women’s exclusive use of hygienic methods in rural India. Additionally, this study explores state- and district-level geographical disparities in the exclusive use of hygienic methods among adolescent women in rural India.

**Methods:**

Information on 95,551 adolescent women from rural India from the latest round of National Family Health Survey (NFHS-5) was analyzed. Bivariate statistics and multilevel logistic regression analysis were used to assess the Individual- and community-level factors associated with exclusive use of hygienic methods among adolescent women in rural India. Choropleth maps were used to discern the geographical disparities in the exclusive use of hygienic methods.

**Results:**

In rural India, only 42% of adolescent women exclusively used hygienic methods, with substantial geographic disparities at the state and district levels. At the state level, the exclusive use of hygienic methods varied from 23% in Uttar Pradesh to 85% in Tamil Nadu. Even greater variation was observed at the district level. There was a clear north-south divide in the exclusive use of hygienic methods among adolescent women in rural India. The results of multilevel logistic regression indicated a considerable amount of variation in the exclusive use of hygienic methods at community level which further reduced when controlled for individual and community-level factors. Rural Indian adolescent women with higher education (AOR:3.20, 95% CI: 2.81–3.64), from general category (AOR: 1.14, 95% CI: 1.07–1.21), with medium mass media exposure (AOR: 1.43, 95% CI: 1.35–1.51), and from richest wealth quintile (AOR: 3.98, 95% CI: 3.69–4.30) were more likely to use hygienic methods exclusively.

**Conclusion:**

Wide differential across biodemographic and socioeconomic groups, and substantial geographic disparities at state- and district-level in the exclusive use of hygienic methods suggests a need to adopt context-specific interventions for adolescent women in rural India. Distribution of subsidized or free menstrual hygiene methods to disadvantaged adolescent women, and in the low-prevalence districts may increase the level of exclusive use of hygienic methods remarkably.

**Supplementary Information:**

The online version contains supplementary material available at 10.1186/s12889-022-14622-7.

## Introduction

Menstruation is a natural biological process which is often recognized as a period of change from adolescence to womanhood [1–4]. Despite this, millions of adolescent women worldwide are denied the right to control their menstrual cycle in a dignified and healthy manner [2]. To ensure healthy and dignified menstruation, Menstrual Hygiene Management (MHM) has received particular attention from the World Health Organization (WHO) and United Nations International Children’s Emergency Fund (UNICEF) [1, 2, 5].

Adolescent women are often inexperienced in MHM [6–8]. They lack adequate and correct knowledge about their bodies, especially the reproductive system and its working, given the social prohibitions on discussing these issues [6]. They also lack the disposable income to buy hygienic menstrual products [9]. Inability to manage menstrual hygiene can have serious consequences for their physical, mental, and emotional health, as well as their social development and educational attainment [2]. Therefore, managing menstrual health and hygiene among adolescent women is a major public health concern for policymakers in low- and middle-income countries, including India [5, 10].

India hosts about one-fifth of the world’s population of adolescent women. Unfortunately, most of them, especially those living in rural areas, typically face many restrictions that limit their agency and autonomy [7]. During menstruation, these restrictions become much more severe, preventing them from participating in many aspects of social life, worshipping, bathing, cooking, and sexual activity [7, 9, 11, 12]. Millions of adolescent girls in India drop out of school every year due to restrictions on mobility, a lack of restrooms and disposal facilities in schools, and fear or shame caused by the odour and stains of menstrual blood [13]. The situation is further worsened by the widespread ignorance around puberty and menstruation, the lack of access to menstrual hygiene products, and the absence of adequate water, sanitation, and hygiene facilities, leading to poor menstrual hygiene practices [14, 15].

Poor menstrual hygiene practices may cause reproductive and urinary tract infections in addition to rashes, itching, foul odour, and many other reproductive health morbidities [16–18]. Poor menstrual hygiene management can also compromise women’s educational and economic opportunities. In addition, several sustainable development goals (SDGs) such as SDG 3 (healthy lives and well-being for all), SDG 4 (inclusive and equitable education), SDG 5 (gender equality), and SDG 8 (equal economic opportunities) cannot be achieved without ensuring safe and dignified menstruation women of all ages [19, 20]. Therefore, it is crucial for the policy makers to understand the access to and use of menstrual hygiene methods among adolescent women, especially in rural areas of India, where a large proportion of the country’s adolescent population resides.

A few studies in recent years have sought to explore the knowledge, attitude, and prevalence of hygienic absorbent use among adolescent girls in India [8, 21–25]. However, most studies have only been carried out in small geographical areas. Though small-scale studies provide valuable insights into people’s health behaviours, their results are not generalizable to a wider population due to insufficient geographic coverage and a small sample size, which limits their power. An increasing amount of literature in the recent past has examined the factors affecting the use of hygienic methods during menstruation among young women (aged 15–24) in India [16, 26–29]. They have identified that the use of hygienic methods during menstruation is associated with level of education, household wealth, mass media exposure, and place of residence [26, 27].

Despite various government schemes to promote menstrual hygiene during menstruation in rural areas by providing subsidized sanitary napkins, data from the most recent round of the National Family Health Survey (NFHS-5) indicate that the use of hygienic methods during menstruation is still lower in rural areas than in urban areas [16, 26–28]. Yet, no study in India has examined how the use of hygienic methods varies among adolescent women in rural India using a nationally representative sample. The review of previous literature also reveals that sub-national geographical disparities in the use of hygienic methods among adolescent women in rural India have remained unexplored [30].

To increase the use of hygienic methods during menstruation among adolescent women in rural India, it is necessary to identify the disadvantaged subsets of this population so that policymakers and programme designers may focus their efforts on them. Therefore, the present study examines the correlates of exclusive use of hygienic methods during menstruation among adolescent women in rural India. In addition, it examines state- and district-level variation in the exclusive use of hygienic methods to provide reliable, research-based evidence on geographic disparity in the use of hygienic methods among rural adolescent women in India.

## Data and methods

**Fig. 1:**
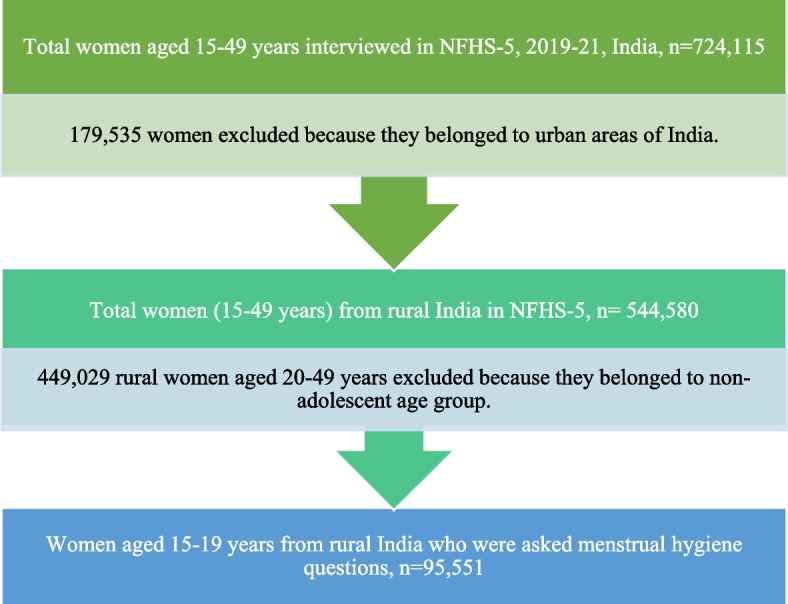
Flow chart showing the steps to select a representative sample of rural Indian adolescent women aged 15–19 for the current study

This study used data from the fifth National Family Health Survey (2019–2021). The NFHS is a nationally representative cross-sectional survey that collects data on various demographic, socioeconomic, maternal and child welfare, reproductive health, and family planning aspects. NFHS-5 interviewed 724,115 women of the reproductive age group (15–49 years) and 101,839 men of age 15–54 years from 636,669 households in 28 states and 8 Union territories (UTs) across 707 districts, with a response rate of 97%. The detailed sampling procedure, sample size, and findings are available in the national report [31]. For this study, data for 95,551 adolescent women aged 15–19 from 28 states and 8 UTs were included in the analysis (see Fig. 1). The NFHS asks women questions on different methods used during menstruation, which is asked to all women, except women aged 25 or above who have had a hysterectomy or have never menstruated. For the current study, methods such as sanitary napkins, tampons, and menstrual cups were considered hygienic [29].

### Conceptual framework

**Fig. 2:**
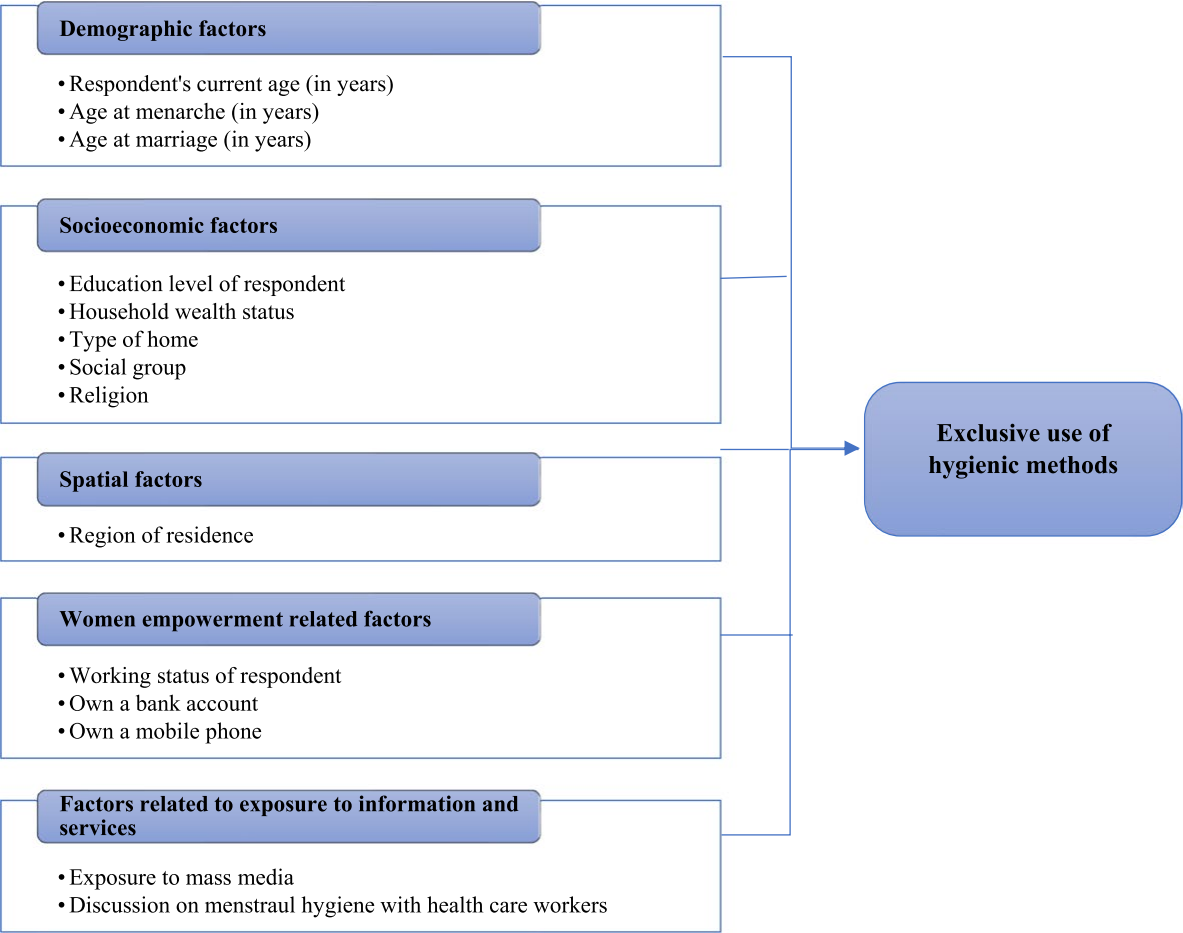
Conceptual Framework: Factors affecting exclusive use of hygienic methods

The analysis for this study is based on the framework adapted from existing literature on hygienic methods during menstruation [15, 19, 24–26]. The framework shows pathways through which various factors might affect the exclusive use of hygienic methods among adolescent women in rural India. It was hypothesized that the exclusive use of hygienic methods was associated with demographic factors, socioeconomic factors, geographic factors, and factors related to exposure to information and services. The list of variables considered for analysis is provided later in this section. The conceptual framework based on which the analysis for this study was conducted is given in Fig. 2.

### Variables

#### Dependent variable

NFHS-5 asks a multiple-response question to eligible female respondents about methods used during their menstrual period to prevent blood stains from becoming evident. Response options included seven categories, i) locally made napkins, ii) sanitary napkins, iii) tampons, iv) menstrual cups, v) cloth, vi) nothing, and vii) others. The NFHS-5 categorizes these methods into two: hygienic and unhygienic. The first four of these are labelled as hygienic methods, and the remaining as unhygienic [16].

The outcome variable of this study is “exclusive use of hygienic methods”. It is a binary variable. A woman is considered “an exclusive user of hygienic methods” if she uses hygienic methods only. This category was coded as ‘1’. Any woman who either uses unhygienic methods or a combination of hygienic and unhygienic methods is considered “not an exclusive user of hygienic methods”. This category was coded as ‘0’. This variable has been defined in this way in many previous studies in India [16, 18, 26, 30, 32].

#### Independent variables

Several relevant socioeconomic and demographic predictors (including respondent’s current age, age at menarche, age at marriage, woman’s education, social group, religion, household wealth status, region of residence, types of home, exposure to mass media, discussion on menstrual hygiene with a healthcare worker, respondent’s working status, and ownership of a bank account and mobile phone were included in the analysis. The independent variables were selected based on previous research on menstrual hygiene management and the availability of variables in the NFHS-5 dataset [15, 25–28]. Table 1 describes the independent variables used in this study in detail.

**Table 1:** Operational definition of variables used in the study

Variables	Description
Individual-level variables	
Age at menarche (in years)	Age at menarche indicates the age of onset of the first menstrual period of a woman. It is divided into four categories- ‘less than 12 years’ (1); ‘13–15 years’ (2); ‘more than 16 years’ (3). Some of them don’t remember the age; they are coded as ‘don’t know’ (0)
Age at marriage (in years)	Age at marriage is divided into 3 categories- ‘marriage before 18 years’ (0), ‘marriage after 18 years’ (1), and ‘not married’ (2)
Respondent’s education	Education level is categorized into four groups- ‘no education’ (0), ‘primary’ (1), ‘secondary’ (2) and ‘higher’ (3)
Household wealth status	The wealth index is a composite index of household amenities and assets; it indicates the socioeconomic condition of a household. In NFHS-5, every household is given a score based on the number of consumer goods they own. A total of 33 assets and housing characteristics were taken into consideration to prepare a factor score using Principal Component Analysis. Thereafter this factor score is divided into five equal categories, − ‘poorest’ (1); ‘poorer’ (2); ‘middle’ (3); ‘richer’ (4); ‘richest’ (5) each with 20% of the population.
Social groups	Social groups are divided into 4 categories- ‘Scheduled Caste’ (1), ‘Scheduled Tribe’ (2), ‘Other Backward Classes’ (3), ‘Others’ (General) (4)
Religion	Religion is divided into four categories – ‘Hindu’ (1); ‘Muslim’ (2); ‘Christian’ (3) ‘Others’ (4). Others include all religious groups other than Hindu, Muslim, and Christian.
Region of residence	To construct this variable, Indian states are grouped into 6 categories. ‘Northern’ (1) includes Jammu & Kashmir, Ladakh, Himachal Pradesh, Punjab, Rajasthan, Haryana, Uttarakhand, Chandigarh (Union Territory - UT) and Delhi; ‘central’ (2) includes the states of Uttar Pradesh, Madhya Pradesh and Chhattisgarh; ‘eastern’ (3) includes the states of Bihar, Jharkhand, West Bengal and Odisha; ‘western’ (4) includes the states of Gujarat, Maharashtra, Goa and UTs of Dadra & Nagar Haveli and Daman & Diu; ‘southern’ (5) includes the states of Kerala, Karnataka, Andhra Pradesh, Tamil Nadu and the UTs of Andaman & Nicobar Islands, Pondicherry and Lakshadweep); ‘north-eastern’ (6) includes the states of Sikkim, Assam, Meghalaya, Manipur, Mizoram, Nagaland, Tripura, and Arunachal Pradesh. This classification has been used by the NFHS-5 report [31]
Type of home	We have recoded the variable v150 (labelled as “relationship to household head” in NFHS-5 ‘individual’ dataset to generate a categorical variable, ‘Type of home’. Based on the respondent’s relationship with the household head, four categories were created, − marital home, natal home, other home, and household head. ‘Marital home’ (1) includes wife, daughter-in-law, parent, parent-in-law, co-spouse, niece by marriage, sister-in-law; ‘natal home’ (2) includes daughter, grandchild, sister, other relatives, niece by blood, niece; ‘other’s home’ (3) includes domestic servant, adopted/foster child, non-relatives; and ‘head of the household’ (4) respondents who are household head themselves [26].
Working status	Working status indicates the employment condition of the respondent. A dichotomous variable is formed: ‘not working’ (1) and ‘working’ (2)
Exposure to mass media	Three questions were asked to women in NFHS-5 survey. They are i) how often they read newspaper/magazines, ii) how often they watch television, and iii) how often they listen to radio. The responses are ‘almost every day’, at least once a week, less than once a week and not at all. Based on these responses a composite index is computed and divided into four categories: ‘no exposure’ (0) if the respondent is not exposed to any mass media; ‘low exposure’ (1) if a respondent is exposed to any one type of mass media; ‘medium exposure’ (2) if the respondent is exposed to any two types of mass media; ‘high exposure’ (3) if the respondent is exposed to all three types of mass media.
Discussed menstrual hygiene with healthcare workers (in last 3 months)	Questions were asked to the respondents in NFHS-5, they are- i) in the last 3 months, if the respondent has met with any health worker- including an auxiliary nurse midwife (ANM), accredited social health activist (ASHA), *Anganwadi* worker (AWW), also known as Integrated Child Development Services worker, multipurpose worker (MPW), or any other community health worker; and ii) if they have discussed about menstrual hygiene during the meeting. If respondent did not discuss menstrual hygiene with healthcare workers, then they are coded as 0, if discussed 1.
Own a bank account	Whether a respondent owns a bank/savings account by herself – ‘yes’ (1); ‘no’ (0)
Own a mobile phone	Whether a respondent owns a bank/savings account by herself – ‘yes’ (1); ‘no’ (0)
Community level variables	
Proportion of women with secondary level of education in PSU	The proportion of women with secondary level of education in PSUs variable has been categorized into three groups, ‘0–25% of women with secondary level of education in PSUs’ (1); ‘26–50% of women with secondary level of education in PSU’ (2); and ‘more than 50% women with secondary level of education in PSU’ (3)
Proportion of poor women in PSU	The proportion of poor women in PSUs variable has been categorized into three groups, ‘0–25% of poor women living in PSUs’ (1); ‘26–50% of poor women living in PSUs’ (2); and ‘more than 50% poor women living in PSUs’ (3)
Proportion of SC/ST women in PSU	The proportion of SC/ST women in PSUs, this variable has been categorized into three groups, ‘0–25% of SC/ST women living in PSUs’ (1); ‘26–50% of SC/ST women living in PSUs’ (2); and ‘more than 50% SC/ST women living in PSUs’ (3)

### Statistical analysis

To begin with, the study used bivariate analysis techniques to underscore the differences in the exclusive use of hygienic methods by adolescent women (aged 15–19 years) of rural India during menstruation by their socioeconomic predictors recorded in the study. To assess the association between outcome and each predictor variable, Chi-square test was employed.

Due to the clustering of individuals within primary sampling units (PSUs), a multilevel statistical model was required for our study [33]. Multilevel modelling controls potential clustering effects and corrects any bias arising out of that in the standard errors [34]. As the nature of our outcome variable was binary, we used a multilevel logistic regression model with two-level to investigate the effect of measurable individual and community level factors (fixed effects) on the exclusive use of hygienic methods [35]. The ‘runmlwin’ command in Stata 16 was used to estimate the random effects at the community level [36].

Two models for the dependent variable were estimated. In the first model, no explanatory variables were included (null/empty model). The final model expanded on the previous model by adding the individual- and community-level variables found statistically significant in the Chi-square test. The fixed-effect (association measures) results are shown as odds ratio (OR) with 95% confidential intervals (CIs). The results of random effects (variation measures) are presented as the variance partition coefficient (VPC) [37].

As the study included various variables that might be correlated, variance inflation factors (VIF) were calculated to assess the multicollinearity. The results of the multilevel logistic regression were presented in the form of adjusted odds ratios and 95% confidence intervals. For statistical analysis and modelling, Stata 16 software was used [38]. The 'Svyset*'* command was used in Stata to adjust for the complex survey design (sampling weights, clustering, and stratification) of the NFHS-5 [39]. ArcMap 10.5 software was used for preparing maps to show the spatial distribution of the outcome variable [40].

## Results

### Respondent characteristics

Table 2 shows the distribution of adolescent women in rural India covered by the study sample by their socio-demographic characteristics. Out of the 95,551 adolescent women in rural India aged 15–19, about 87% of women were unmarried, and most women had their menarche between 13 and 15 years of age. About two-thirds of women were Hindu, and about two-fifths belonged to Other Backward Classes (OBCs). Only about a quarter of sampled women reported having high mass media exposure. About half of the respondents lived in the central and east regions of the country.

**Table 2:** Percentage distribution of adolescent women by background characteristics in rural India, NFHS-5 (2019–21)

Background characteristics	N (95,551)	%
**Individual-level variables**		
**Age at menarche (in years)**		
Do not remember	390	0.4
≤ 12	19,057	19.9
13–15	72,836	76.2
≥ 16	2595	2.7
Missing	673	0.7
**Age at marriage (in years)**		
Not married	82,924	86.8
< 18 years	7210	7.6
≤ 18 years	5384	5.6
Do not know	33	0.0
**Respondent’s education**		
No education	4765	5.1
Primary	5556	5.8
Secondary	80,083	83.8
Higher	5147	5.4
**Religion**		
Hindu	73,119	76.5
Muslim	12,172	12.7
Christian	6212	6.5
Others	4048	4.2
**Social group**		
Do not know	408	0.4
Scheduled Caste	20,002	20.9
Scheduled Tribe	19,634	20.6
Other Backward Classes	37,146	38.9
Others	13,939	14.6
Missing	4422	4.6
**Household wealth**		
Poorest	19,802	20.7
Poorer	21,892	22.9
Middle	20,547	21.5
Richer	18,018	18.9
Richest	15,292	16.0
**Region of residence**		
North	17,907	18.7
Central	27,819	29.1
East	19,139	20.0
West	7612	8.0
Southern	9842	10.3
North-east	13,232	13.9
**Type of home**		
Marital	9439	9.9
Natal	85,515	89.5
Other	278	0.3
Head of the household	319	0.3
**Exposure to mass media**		
No exposure	22,449	23.5
Low exposure	45,816	48.0
Medium exposure	23,723	24.8
High exposure	3563	3.7
**Discussed menstrual hygiene with a healthcare worker**		
No	93,401	97.8
Yes	2150	2.2
**Working status**		
Question not asked	81,361	85.2
Not working	12,402	13.0
Working	1788	1.8
**Owns a bank account**		
Question not asked	81,361	85.2
No	4858	5.1
Yes	9332	9.7
**Owns a mobile phone**		
Question not asked	81,361	85.2
No	9842	10.3
Yes	4348	4.5
**Community-level variables**		
**Proportion of women with secondary education in PSU**		
0–25%	28,049	29.3
26–50%	30,533	32.0
> 50%	36,969	38.7
**Proportion of poor women in PSU**		
0–25%	37,098	38.8
26–50%	20,598	21.6
> 50%	37,855	39.6
**Proportion of SC/ST women in PSU**		
0–25%	39,294	41.9
26–50%	19,677	21.0
> 50%	34,795	37.1

Note: *N* number of adolescent women in rural India, % weighted percentage of N

### Exclusive use of hygienic methods by background characteristics

About 42% of adolescent women in rural India reported exclusive use of hygienic methods during menstruation. Table 3 shows the proportion of adolescent women using only hygienic methods by background characteristics in rural India. The use of hygienic methods was slightly higher in adolescents who had menarche after age 16 than in women who had menarche before age 16. The exclusive use of hygienic methods was relatively higher among those married after the legal age of 18 years (47%). The exclusive use among those with higher education was four times higher (62%) than those without education (15%). Further, the exclusive use of hygienic methods was considerably higher among Christians (56%) than Hindus (43%) and Muslims (35%). Only 37% of Scheduled Tribe women reported exclusive use of hygienic methods compared with 51% of General (Others) category women.

**Table 3:** Percentage of adolescent women in rural India who exclusively used hygienic methods during menstruation, by selected background characteristics, NFHS-5 (2019–21)

Background characteristics	No. of women exclusively using hygienic methods [N, weighted]	Percent of women exclusively using hygienic methods (weighted percentage)	
		Yes	No
**Individual level variables**			
**Age at menarche (in years)**		**χ**^**2**^ **= 84.14,** ***p*** **< 0.001**	
≤ 12	7368	44.7 [43.4,45.9]	55.3 [54.1,56.6]
13–15	31,986	42.3 [41.7,43.0]	57.7 [57.0,58.4]
≥ 16	1240	49.4 [46.9,51.9]	50.6 [48.1,53.2]
**Age at marriage (in years)**		**χ**^**2**^ **= 67.93, p < 0.001**	
Not marriage	34,339	42.5 [41.9,43.2]	57.5 [56.8,58.1]
< 18 year	3515	40.1 [38.4,41.9]	59.8 [58.2,61.6]
≥ 18 year	2813	46.5 [44.8,48.3]	57.5 [51.7,55.3]
**Respondent’s education**		**χ**^**2**^ **= 3784.92, p < 0.001**	
No education	746	15.0 [13.7,16.4]	85.0 [83.7,86.3]
Primary	1088	19.4 [18.0,20.9]	80.6 [79.1,82.0]
Secondary	35,302	44.5 [43.9,45.2]	55.5 [54.8,56.1]
Higher	3536	62.0 [60.1,63.8]	38.0 [36.2,39.9]
**Religion**		**χ**^**2**^ **= 726.11, p < 0.001**	
Hindu	33,767	43.0 [42.3,43.6]	43.0 [56.4,57.7]
Muslim	4630	35.4 [33.7,37.1]	64.6 [63.0,66.3]
Christian	948	55.5 [52.2,58.8]	44.5 [41.2,47.8]
Others	1326	61.9 [59.2,64.5]	38.1 [35.5,40.8]
**Social groups**		**χ**^**2**^ **= 669.44, p < 0.001**	
Scheduled Caste	9734	42.4 [41.3,43.5]	57.6 [56.5,58.8]
Scheduled Tribe	4076	36.6 [35.2,38.0]	63.4 [62.0,64.8]
Other Backward Classes	17,363	41.3 [40.4,42.1]	58.7 [57.9,59.6]
Others	7442	51.2 [49.8,52.6]	48.8 [47.4,50.2]
**Household wealth status**		**χ**^**2**^ **= 7990.03, p < 0.001**	
Poorest	4864	24.4 [23.4,25.4]	75.6 [74.6,76.6]
Poorer	6938	32.4 [31.4,33.3]	67.7 [66.7,68.6]
Middle	8766	43.1 [42.0,44.2]	56.9 [55.8,58.0]
Richer	10,068	54.6 [53.5,55.7]	45.4 [44.3,46.5]
Richest	10,036	65.4 [64.2,66.6]	34.6 [33.4,35.8]
**Region of residence**		**χ**^**2**^ **= 9356.93, p < 0.001**	
North	7321	58.2 [56.8,59.6]	41.8 [40.4,43.2]
Central	7563	24.9 [24.1,25.7]	75.1 [74.3,75.9]
East	10,849	40.6 [39.3,41.9]	59.4 [58.1,60.7]
West	5254	56.2 [54.4,58.1]	43.8 [41.9,45.7]
South	8519	67.5 [65.7,69.2]	32.5 [30.8,34.3]
North-east	1165	30.1 [28.5,31.8]	69.9 [68.2,71.5]
**Type of home**		**χ**^**2**^ **= 18.86,** ***p*** **< 0.017**	
Marital	4803	44.3 [42.8,45.7]	55.8 [54.3,57.2]
Natal	35,690	42.4 [41.7,43.0]	57.6 [57.0,58.3]
Other	59	39.0 [30.7,48.0]	61.0 [52.0,69.3]
Head of the household	119	36.9 [29.9,44.5]	63.1 [55.5,70.2]
**Exposure to mass media**		**χ**^**2**^ **= 4628.47, p < 0.001**	
No exposure	5969	25.5 [24.6,26.5]	74.5 [73.6,75.4]
Low exposure	19,866	43.8 [43.0,44.5]	56.2 [55.5,57.0]
Medium exposure	12,921	55.3 [54.3,56.3]	44.7 [43.7,45.7]
High exposure	1916	56.7 [54.3,59.1]	43.3 [40.9,45.7]
**Discussed menstrual hygiene with healthcare worker**		**χ**^**2**^ **= 74.28, p < 0.001**	
No	39,531	42.4 [41.0,43.7]	57.7 [57.0,58.3]
Yes	1140	51.5 [48.5,54.6]	48.5 [45.4,51.5]
**Working status**		**χ**^**2**^ **= 53.52 p < 0.001**	
Not working	5350	42.5 [41.1,44.0]	57.8 [56.0,58.9]
Working	563	33.8 [30.7,37.0]	66.2 [63.0,69.3]
**Respondent owns a bank account**		**χ**^**2**^ **= 157.30,** p **< 0.001**	
No	1591	34.2 [32.2,36.3]	65.8 [63.7,67.8]
Yes	4322	45.0 [43.4,46.7]	55.0 [53.3,56.6]
**Respondent owns a mobile phone**		**χ**^**2**^ **= 199.93, p < 0.001**	
No	3935	38.0 [36.4,39.6]	62.0 [60.4,63.6]
Yes	1977	50.9 [48.6,53.2]	49.1 [46.8,51.5]
**Community level variables**			
**Proportion of women with secondary level of education in PSU**		**χ**^**2**^ **= 4842.97, p < 0.001**	
0–25%	7978	72.0 [71.0,73.0]	28.0 [27.0,29.0]
26–50%	11,983	60.9 [59.8,61.9]	39.1 [38.1,40.2]
> 50%	20,272	45.1 [44.1,46.1]	54.9 [54.0,55.9]
**Proportion of poor women in PSU**		**χ**^**2**^ **= 5589.15, p < 0.001**	
0–25%	21,148	43.2 [42.2,44.2]	56.8 [55.9,57.8]
26–50%	8330	59.5 [58.3,60.8]	40.5 [39.2,41.7]
> 50%	10,755	70.1 [69.2,71.0]	29.9 [29.0,30.8]
**Proportion of SC/ST women in PSU**		**χ**^**2**^ **= 14.03,** ***p*** **= 0.1672**	
0–25%	16,985	56.8 [55.8,57.7]	43.2 [42.3,44.2]
26–50%	8272	58.3 [57.0,59.6]	41.7 [40.4,43.0]
> 50%	14,471	57.3 [56.2,58.4]	42.7 [41.6,43.8]
**Total**	**40,223**	**57.4**	**42.6**

Note: Figures in parentheses are 95% confidence interval; **χ**^**2**^ test applied for each variable, PSU: primary sampling units

Only 24% of women in the lowest wealth quintile reported exclusive use of hygienic methods. In comparison, 65% of women in the highest wealth quintile did so. Furthermore, the percentage of women who reported exclusive use of hygienic methods was 57% among those who were fully exposed to mass media compared to 26% among those who were not. The exclusive use was higher among those women who met and discussed menstrual hygiene issues with healthcare workers in the three months prior to the survey than those who did not (52% vs 42%).

The exclusive use of hygienic methods was higher among those women who owned a bank account and mobile phone than those who did not. In addition, the exclusive use of hygienic methods was higher in the rural areas of southern (68%), northern (58%), and western regions (56%) of India as compared to the rural areas of central and north-eastern regions (25 and 30% respectively).

The findings also show significant diversity in the exclusive use of hygienic methods at the regional, state-, and district-levels (described below).

### Spatial patterns of exclusive use of hygienic methods among adolescent women across 28 states, 8 UTs, and 707 districts of India

Analysis at the regional level provides only a broad idea regarding spatial variation in the exclusive use of hygienic methods. It marks spatial heterogeneity at the macro level. Therefore, we mapped the exclusive use of hygienic methods at the state and district level of India.

**Fig. 3:**
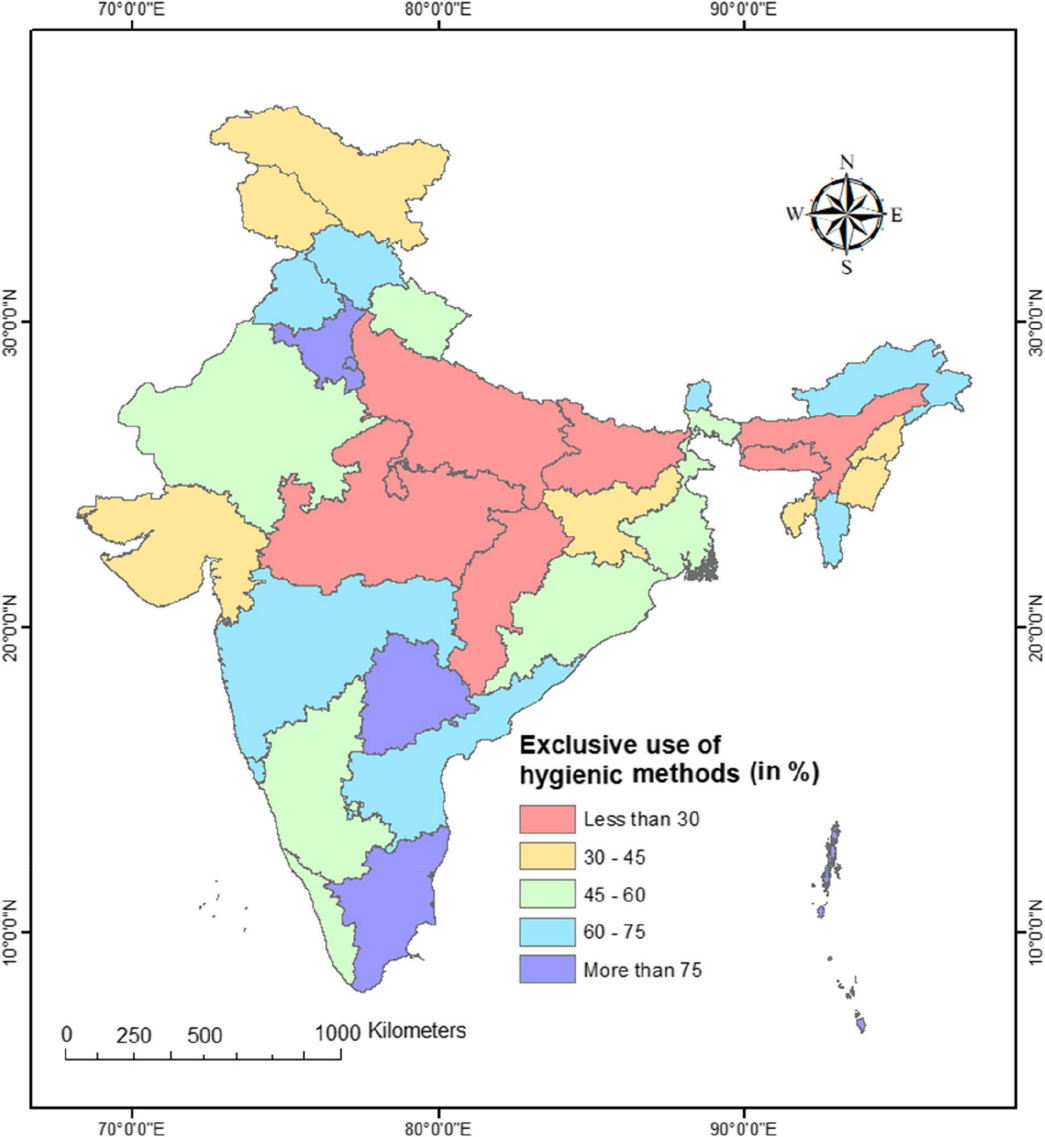
State-wise distribution of exclusive use of hygienic methods during menstruation among women aged 15–19 in rural India, NFHS-5, 2019–21

Figure 3 indicates a substantial state-wise variation in the exclusive use of hygienic methods among adolescent women in rural India. Among the 28 states, Uttar Pradesh (24%) had the lowest prevalence of exclusive use of hygienic methods, followed by Madhya Pradesh (26%), Bihar (29%), Chhattisgarh (29%), and the north-eastern state of Assam (29%). On the other hand, exclusive use of hygienic methods was highest in Tamil Nadu (85%), followed by Telangana (82%) among states and Andaman and Nicobar (92%), followed by Puducherry (91%) among Union Territories. On the other hand, the prevalence of exclusive use of hygienic methods was modest in Gujarat (41%) and Kerala (56%).

**Fig. 4:**
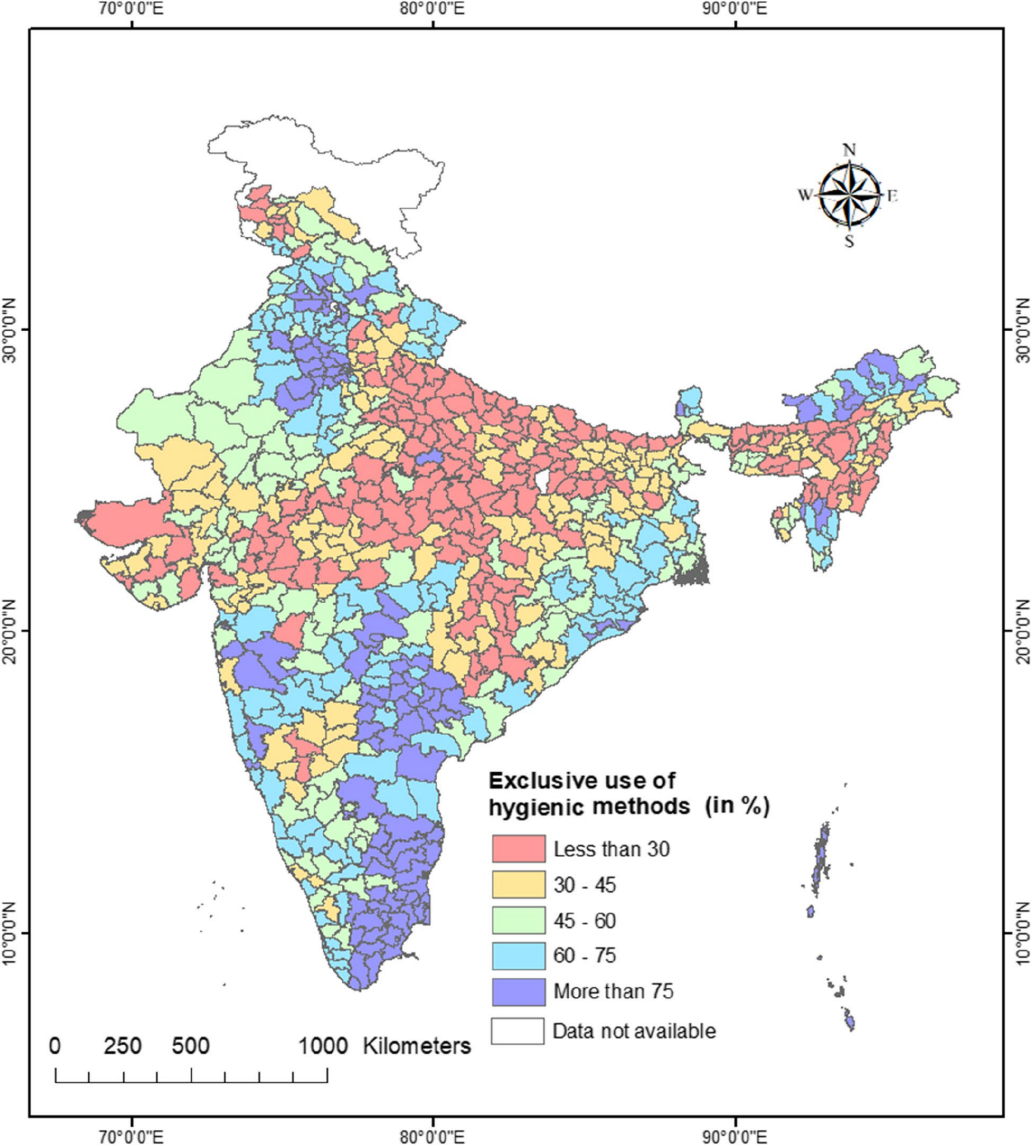
District-wise distribution of exclusive use of hygienic methods during menstruation among women aged 15–19 years in rural India, NFHS-5, 2019–21

Figure 4 depicts the district-level spatial pattern of exclusive use of hygienic methods among adolescent women. The geographical pattern of adolescent women’s exclusive use of hygienic methods at the district-level is considerably more varied than the state-level geographical pattern. The exclusive use of hygienic methods ranged from 6 to 8% in the West Jaintia Hills and West Khasi Hills districts of Meghalaya to 100% in the Thoothukuddi and Kanyakumari districts of Tamil Nadu. The state average obscures any differences between individual districts within a state. Therefore, we analysed the patterns at the district level. The analysis of within-state district-level patterns revealed considerable between-district variation in the exclusive use of hygienic methods in many states. In Uttar Pradesh, for instance, the exclusive use of hygienic methods ranged from 7% in Banda to 45% in Goutam Buddha Nagar. In Madhya Pradesh, the exclusive use of hygienic methods ranged from 8% in Umaria and Sidhi to 63% in Balaghat. Similarly, the exclusive use of sanitary methods in Tamil Nadu ranged from 47% in Erode to 100% in Thoothukuddi and Kanyakumari. In Karnataka, it ranged from 25% in Gadag and Bagalkot to 75% in Udupi and Kolar districts.

In almost a quarter of all districts (179 out of 707), the exclusive use of hygienic methods was less than 30%. Three distinct pockets of low exclusive use of hygienic methods can be identified. The first pocket is spread over large swathes of central Indian states of Uttar Pradesh, Madhya Pradesh, Bihar, and Chhattisgarh. The second pocket is located in northeast India, comprising the districts of Assam, Nagaland, Manipur, and Meghalaya. The third pocket comprises the districts from western Gujarat, northern Karnataka, and Jammu & Kashmir. There were multiple pockets of ultra-low exclusive use of hygienic procedures among these pockets of low exclusive use of hygienic methods, with fewer than 15% of women reporting exclusive use of hygienic methods. For example, in Madhya Pradesh (MP), there were two such pockets – one in the eastern MP comprising Barwani, Jhabua and Alirajpur districts, and the other in the western MP comprising Sidhi, Umaria, Damoh, and Dindori districts.

About 17% of all districts in the country (111 out of 707) had a prevalence of over 75%. Thus, there were three main pockets of high exclusive use of hygienic methods in the country – a) the southern pocket included districts from Tamil Nadu, Andhra Pradesh, Telangana, and Maharashtra b) the northern pocket included districts of Haryana, Punjab and Himachal Pradesh, c) the north-eastern pocket included districts from Arunachal Pradesh and Mizoram.

### Determinants of exclusive use of hygienic methods

The determinants of the exclusive use of hygienic methods were examined using a multilevel logistic regression model. Before fitting the multilevel model, we assessed the association between the exclusive use of hygienic methods and potential individual- and community-level independent variables using the Chi-squared test. It was done to eliminate any variables not associated with the dependent variable. Only those variables which were found statistically significant (*p* ≤ 0.05) were included in the final logistic regression model. In addition, we also calculated variance inflation factors (VIFs) to check the degree of multicollinearity between the independent variables included in the regression model. Since all the variables had a VIF within the acceptable range of 5 [41], we did not have to modify or remove from the final regression model any of the variables that we began our analysis with (for detailed VIF values, see supplementary Table 1, Additional file 1).

The first step of applying any multilevel model is determining whether the data supports the decision to assess random effects at higher levels. In this study, we set up a two-level random intercept-only model (a model with no covariates, also known as the null model). The variance partition coefficient (VPC) revealed that about 39% of the total variance in the exclusive use of hygienic methods was attributable to the differences across communities (see Table 4).

**Table 4:** Variance coefficients corresponding to the null model

Random effects	Exclusive use of hygienic methods
Community (PSU) random variance (SE.)	2.144 (0.034)
Community (PSU) VPC (in %)	35

Note: *SE* standard error, *VPC* variance partition coefficient

Table 5 presents the odds ratios obtained from the two-level logistic regression fitted to examine the factors affecting the exclusive use of hygienic methods among adolescent women in rural India. The random parameters revealed that the exclusive use of hygienic methods varied considerably at the community level, however, after controlling for the community-level factors, the variation in the use of hygienic methods attributed to the differences across communities reduced from 35 to 30%.

**Table 5:** Results of multilevel logistic regression showing the factors associated with exclusive use of hygienic methods among adolescent women in rural India NFHS-5 (2019–21)

Variables	Adjusted odds ratio	***p***-value	95% CI	
			Lower	Upper
**Fixed effects**				
***Individual-level variables***				
**Age at menarche (in years)**				
≤ 12®				
13–15	1.07	0.003	1.02	1.12
≥ 16	1.57	< 0.001	1.41	1.76
**Age at marriage (in years)**				
Not marriage®				
< 18 year	0.86	0.002	0.78	0.95
≥ 18 year	1.06	0.250	0.96	1.18
**Respondent’s education**				
No education®				
Primary	1.28	< 0.001	1.12	1.45
Secondary	2.48	< 0.001	2.23	2.75
Higher	3.20	< 0.001	2.81	3.64
**Religion**				
Hindu®				
Muslim	0.62	< 0.001	0.58	0.67
Christian	1.11	0.050	1.00	1.24
Others	1.44	< 0.001	1.30	1.59
**Social group**				
Scheduled Caste®				
Scheduled Tribe	1.03	0.452	0.96	1.10
Other Backward Classes	0.96	0.109	0.91	1.01
Others	1.14	< 0.001	1.07	1.21
**Household wealth**				
Poorest®				
Poorer	1.32	< 0.001	1.25	1.40
Middle	1.80	< 0.001	1.69	1.92
Richer	2.43	< 0.001	2.27	2.61
Richest	3.98	< 0.001	3.69	4.30
**Region of residence**				
Central®				
North	3.42	< 0.001	3.17	3.70
East	3.22	< 0.001	2.99	3.46
West	3.20	< 0.001	2.91	3.52
South	6.45	< 0.001	5.90	7.06
North-east	2.76	< 0.001	2.51	3.04
**Type of home**				
Marital®				
Natal	0.91	0.059	0.83	1.00
Other	0.66	0.014	0.47	0.92
Head of the household	0.95	0.731	0.70	1.29
**Exposure to mass media**				
No exposure®				
Low exposure	1.26	< 0.001	1.20	1.32
Medium exposure	1.43	< 0.001	1.35	1.51
High exposure	1.35	< 0.001	1.22	1.49
**Discussed menstrual hygiene with a healthcare worker**				
No®				
Yes	1.01	0.824	0.90	1.14
**Working status**				
Not working®				
Working	0.75	< 0.001	0.66	0.87
**Owns a bank account**				
No®				
Yes	1.06	0.269	0.96	1.17
**Owns a mobile phone**				
No®				
Yes	1.25	< 0.001	1.13	1.38
***Community-level variables***				
**Proportion of women with secondary education in PSU**				
0–25%®				
26–50%	1.33	< 0.001	1.24	1.41
> 50%	1.68	< 0.001	1.57	1.79
**Proportion of poor women in PSU**				
0–25%®				
26–50%	0.89	0.001	0.83	0.95
> 50%	0.76	< 0.001	0.71	0.81
**Random effects**				
Community (PSU) random variance (SE.)		1.393 (0.286)		
Community (PSU) VPC (%)		30.0		

Notes: *CI* confidence interval,® reference category, *PSU* primary sampling units, *SE* standard error, *VPC* variance partition coefficient

Results of the multilevel model show that the odds of exclusive use of hygienic methods in women who were married off before the legal age of 18 years were 14% lower (AOR: 0.86, 95% CI: 0.78–0.95) than the unmarried women (see Table 5). Women with secondary education were about two and a half times (AOR: 2.48, 95% CI: 2.23–2.75), and women with higher education were about three times (AOR:3.20, 95% CI: 2.81–3.64) more likely to use hygienic methods. Muslim women were almost 40% less likely (AOR: 0.62, 95% CI: 0.58–0.67) to exclusively use hygienic methods than their Hindu counterparts. The odds of exclusive use of hygienic methods were higher among the general category (AOR: 1.14, 95% CI: 1.07–1.21) than among SCs.

Wealth status was positively associated with the exclusive use of hygienic methods among adolescent women in rural India. Women from the wealthiest quintile were nearly four times (AOR: 3.98, 95% CI: 3.69–4.30) more likely to use hygienic methods exclusively than women from the poorest quintile. In addition, women with medium exposure to mass media were 43% more likely to use hygienic methods (AOR: 1.43, 95% CI: 1.35–1.51) than women without mass media exposure.

The odds of exclusive use of hygienic methods varied significantly across regions of India. Adolescent women of the southern region (AOR: 6.45, 95% CI: 5.90–7.06) were more likely to use hygienic methods exclusively than those of the central region.

At the community level, the proportion of women with secondary education in a village was positively associated with the exclusive use of hygienic methods during menstruation. On the other hand, an increase in the proportion of poor women in PSUs was associated with decreased odds of exclusive use of hygienic methods.

## Discussion

The present study examined individual and community-level factors associated with the exclusive use of hygienic methods among adolescent women in rural India. A little over two-fifths of adolescent women in rural India reported exclusive use of hygienic methods, considerably lower than their urban counterparts. The multilevel model identified important individual- and community-level predictors of the exclusive use of hygienic methods among adolescent women in rural India. The multilevel model also demonstrated significant community-level variation in the exclusive use of hygienic methods. Community-level variables used in this study were able to explain some of this variation. It suggests the need to look beyond individual-level factors when examining the exclusive use of hygienic methods during menstruation among adolescent women in rural India. Statistically significant individual-level predictors of exclusive use of hygienic methods among adolescent women in rural India included age at marriage, education, religion, wealth, region of residence, mass media exposure, working status, and mobile ownership.

Similar to many previous studies, this study also found a positive association between the exclusive use of hygienic methods and the age at marriage [16, 26, 27, 42, 43]. It may be because older adolescents have more knowledge about hygiene methods, more flexibility to make their own decisions, and more disposable income to spend on the hygiene products they want [16].

One of the most critical predictors for the exclusive use of hygienic methods among adolescent women of rural areas was their level of education. Compared to uneducated women, adolescent women with higher education were more inclined to adopt hygienic methods [17, 42]. Education confers decision-making autonomy, financial independence, increased knowledge of the benefits of using hygienic methods, and awareness of the risks associated with unclean menstruation practices on women [5, 26, 27]. This finding is in line with previous research on this issue [5, 16, 18, 26, 27].

Religion also turned out to be a predictor of the exclusive use of hygienic methods among adolescent women in rural India. The exclusive use among Muslim adolescent women was lower than among Hindu adolescent women. Many previous studies on this issue have concluded the same [5, 16, 18, 26, 28, 44]. Low decision-making and mobility autonomy, as well as a lack of awareness regarding the availability of hygienic methods, may be at the root of this behaviour among Muslim adolescent girls in rural India [26, 45]. Another possible explanation for the relatively low rate of exclusive use of hygienic methods among Muslim women is that a disproportionate number of them belong to lower socioeconomic strata and have low social status and limited decision-making autonomy within their own households [46].

Household wealth, a proxy for the family’s income, also determined whether or not adolescent women in rural India used hygienic methods exclusively. As household wealth increased, so did women’s exclusive use of hygienic methods. This finding is consistent with other studies that have shown that affluent women are more likely than their less privileged counterparts to use hygienic methods exclusively [5, 26, 27]. Poverty has also been identified as a factor behind unsanitary menstrual behaviours in many previous studies [25, 27, 47, 48]. A pack of 10 sanitary napkins in India currently costs around 30–40 INR (0.39–0.52 USD), which is extremely expensive and unaffordable for most rural households in India [49]. The inability to afford sanitary methods may be one of the reasons why poor women resort to using rags/clothes or other unhygienic methods.

The findings of this study indicate that the exclusive use of hygienic methods rose in tandem with the respondents’ exposure to the media. This finding is in line with many previous studies in India. Mass media helps disseminate appropriate information about innovations to the masses and helps it diffuse and spread to rural areas [5, 26]. It appears to be true with regard to menstrual hygiene practices as well. Mothers aside, mass media are often the primary sources of information about menstrual hygiene among adolescent girls. It broadens their knowledge of the variety of low-cost, subsidized, or free menstrual hygiene methods available and the health advantages of using them [5, 27, 50].

Our study revealed that mobile phone ownership was positively associated with the exclusive use of hygienic methods during menstruation. Adolescent women today benefit from increased mobile phone ownership in various ways, including easy access to information. It is critical to have access to accurate and sufficient information to raise awareness about menstrual hygiene management. In addition, mobile phone availability enables peers to offer each other advice on menstrual hygiene and share their experiences. Many public health interventions in India (Kilkari, Mobile Vani, and Mobile Academy) and elsewhere have used mobile phone messaging services to disseminate health information and improve health behaviour and service delivery [51, 52]. The success of such interventions suggests that the recent rapid and high penetration of mobile phones in rural areas can be leveraged to relay accurate information on menstrual hygiene practices to enhance awareness among adolescent girls in rural India [16].

The prevalence of exclusive use of hygienic methods during menstruation varied greatly across India’s regions, states and districts. Adolescent women of southern and western regions were more likely to use hygienic methods exclusively than the country’s eastern and central regions. These results are consistent with prior research on the subject [16, 26, 27]. One probable explanation for the higher rates of hygienic practises in the southern region is the availability of toilet facilities and separate enclosed spaces for disposing of sanitary napkins in the schoolyard, as found in previous studies [23, 26, 53]. Furthermore, most southern states have a highly functional public healthcare system that has adopted numerous measures, including subsidized sanitary napkin distribution projects, to enhance women’s menstrual health [54].

Tamil Nadu, Kerala, Karnataka, Andhra Pradesh, and Telangana state governments have implemented various free or subsidized sanitary napkin distribution initiatives. In 2011, the Government of Tamil Nadu began providing 20 free sanitary napkins to adolescent women in rural areas of the state through a programme called *Pudhu Yugam* (New Era) [55]. In addition, schools in some areas of these states have placed sanitary napkin vending machines in collaboration with local non-governmental organizations (NGOs), which dispense locally-produced napkins at a reduced price [19, 56]. To ensure that girls between the ages of 10 and 19 always have access to sanitary products, the state government of Karnataka has recently launched the *Shuchi* scheme [57]. Similarly, the state government of Andhra Pradesh has decided to distribute for free ten sanitary napkins per month to all girls in classes seven through 12 as part of its *Swechha* programme [58].

Our study revealed that exclusive use of hygienic methods was particularly low in the central and eastern regions of the country. These regions are characterized by low socioeconomic development, high levels of poverty, and an inefficient healthcare system. These reasons, coupled with the strong presence of social taboos, could be behind the lower exclusive use of hygienic methods among adolescent women in these regions [26, 27]. About 25% of districts reported less than 30% exclusive use of hygienic methods among adolescent women in rural India. These districts were home to almost one-third of the sampled adolescent women. Therefore, it is crucial to focus on these districts if the overall level of exclusive use of hygienic methods among adolescent women in rural India is to be increased. Within the states with low exclusive use of hygienic methods, there were also pockets of districts with ultra-low exclusive use (less than 15%). However, more research is required to discern why these districts lag behind their neighbouring districts despite being in the same state and governed by the same policies.

The state governments of Uttar Pradesh (*Kishori Suraksha Yojna*), Madhya Pradesh (*Udita Yojana*), Bihar (*Kishori Shakti Yojana*), Maharashtra (*Asmita*), Rajasthan (*Udaan*), and Tripura (*Kishori Suchita Abhiyaan*) have implemented several schemes to distribute free or subsidised sanitary napkins in schools [58–63]. However, to date, many of these initiatives are in pilot phases and are yet to be scaled up. Moreover, existing schemes in most states are plagued by a plethora of issues, including procurement and supply issues, poor quality of pads, lack of awareness and knowledge about the schemes, and unaffordable prices [64–66]. This might explain why the exclusive use of hygienic methods is so low and unequal in these states.

Under the National Health Mission (NHM), the Central Government of India has made several efforts to educate young women on the importance of menstrual hygiene and promote the use of hygienic methods during menstruation [67]. To reduce access and cost barriers to sanitary napkin use in rural areas, accredited social health activists (ASHAs) are mandated to sell NHM’s subsidized sanitary napkin brand, ‘Free Days’ at Rs.1 per napkin, to adolescent women [68]. However, this initiative has been hampered by procurement and supply issues, high costs, and a lack of enthusiasm among ASHAs. This may explain why the exclusive use of hygienic methods remains low in many districts across the country [69]. More research is needed to unearth why ASHAs have had less success in promoting hygienic methods. In 2020, the Central Government launched 100% biodegradable sanitary napkins under the brand ‘*Suvidha*’. It was made available at *Jan Aushadhi Kendras* (government-run pharmacies) at a subsidized price. However, as of December 2021, there are only 8640 pharmacies in 707 districts, i.e., about 12 pharmacies per district. Moreover, they are primarily concentrated in metropolitan areas, leaving large swaths of the country’s rural areas unserved [70]. Consequently, the availability and affordability of hygienic methods in rural areas remain persistent problems even today [2, 71].

The major strength of this study is that this is the first national-level study to assess adolescent women’s exclusive use of hygienic methods in rural India, using the latest NFHS-5 data. In addition, it has used a multilevel approach that is appropriate to model hierarchical data, such as that of NFHS-5. Finally, this study also examined in detail the geographical patterns of exclusive use of hygienic methods among adolescent women in rural India, which could help formulate location-specific interventions to enhance the level of exclusive use of hygienic methods.

The study has some limitations despite providing detailed information on the factors influencing adolescent women’s exclusive use of hygienic methods during menstruation in rural areas. First, this study could not establish the causal relationship between predictors and outcome variable, as it needs experimental data to do that. NFHS-5 provides cross-sectional data which can only establish the association between regressed and regressor variables. Second, due to data constraints, we were unable to include certain supply-side variables in our study that, according to the demand-supply framework of healthcare use, are important in explaining the degree of utilization of a service or product. The variables related to the supply of subsidized methods to rural health workers, availability and price of hygienic methods at rural pharmacies and provision stores, and supply situation of sanitary napkins at schools have not been included in the analysis due to the lack of these variables in the NFHS dataset. Among other variables we could not include due to the lack of such variables in the NFHS dataset were social taboos/cultural norms, the sanitary situation at schools and disability issues.

## Conclusion

The exclusive use of hygienic methods is still quite low among adolescent women in rural India. This study highlighted significant differences in adolescent women’s exclusive use of hygienic methods throughout rural India’s regions, states, and districts. We found substantial north-south disparities in the exclusive use of hygienic methods, where the hygienic methods use is considerably low in the central districts of rural India. Improved menstrual hygiene and health has been a part of policy discussions in India for long but the discussion has largely been blind to existing geographical variations in the patterns of the same. This suggests that the future interventions and programmes to enhance menstruation hygiene among adolescent women would focus on reducing geographical differences in rural India. Results of multilevel model revealed that household wealth, education, and mass media exposure were the most important factors associated with the exclusive use of hygienic methods, therefore empowering adolescent women and promoting their education would yield greater results in increasing the level of exclusive use of hygienic methods in rural India. Policymakers and stakeholders could target disadvantaged groups of adolescent women to improve the level of hygienic methods’ exclusive use among them and make the overall progress more equitable. Several government programs have begun in recent years, and these could be supported, expanded and broadened to cover the entire adolescent women population. Furthermore, this study suggests state- and district-specific menstrual hygiene policies to improve the universal access to hygienic methods among adolescent women in rural India.

## Supplementary Information

**Additional file 1: Supplementary table 1.** Variance inflation factors.

## Data Availability

The study utilizes secondary sources of data that are freely available in the public domain through https://dhsprogram.com/methodology/survey/survey-display-541.cfm. Those who wish to access the data may register at the above link and thereafter can download the required data free of cost.
