# Supplementary material for: Menstrual hygiene practices among adolescent women in rural India: a cross-sectional study
Source: BMC Public Health. 2022 Nov 19;22:2126. doi: 10.1186/s12889-022-14622-7 (PMC9675161; doi:10.1186/s12889-022-14622-7)
Supplement: Supplementary file 1 — Additional file 1: Supplementary table 1. Variance inflation factors. [file 12889_2022_14622_MOESM1_ESM.docx]

**Supplementary table 1: Variance inflation factors**

| **Variables** | **VIF** | **1/VIF** |
| --- | --- | --- |
| **Individual level variables** | |  |
| **Age at marriage (in years)** | |  |
| Not marriage® |  |  |
| <18 year | 2.04 | 0.49 |
| >=18 year | 1.94 | 0.52 |
| **Respondent's highest level of education** | | |
| No education® |  |  |
| Primary | 2.05 | 0.49 |
| Secondary | 3.25 | 0.31 |
| Higher | 2.29 | 0.44 |
| **Religion** |  |  |
| Hindu® |  |  |
| Muslim | 1.13 | 0.89 |
| Christian | 1.72 | 0.58 |
| Others | 1.14 | 0.88 |
| **Social groups** |  |  |
| Scheduled Caste® |  |  |
| Scheduled Tribe | 1.02 | 0.98 |
| Other Backward Classes | 1.94 | 0.52 |
| Others | 1.78 | 0.56 |
| **Household wealth status** | 1.6 | 0.63 |
| Poorest® |  |  |
| Poorer | 1.76 | 0.57 |
| Middle | 2.3 | 0.43 |
| Richer | 2.51 | 0.40 |
| Richest | 2.66 | 0.38 |
| **Region of residence** |  |  |
| Central® |  |  |
| North | 1.57 | 0.64 |
| East | 1.41 | 0.71 |
| West | 1.24 | 0.81 |
| South | 1.43 | 0.70 |
| North-east | 1.97 | 0.51 |
| **Type of home** |  |  |
| Marital® |  |  |
| Natal | 2.93 | 0.34 |
| Other | 1.09 | 0.92 |
| Head of the household | 1.06 | 0.95 |
| **Exposure to mass media** | |  |
| No exposure® |  |  |
| Low exposure | 1.84 | 0.54 |
| Medium exposure | 2 | 0.50 |
| High exposure | 1.22 | 0.82 |
| **Responded discussed menstrual hygiene with healthcare worker** | | |
| No® |  |  |
| Yes | 1.01 | 0.99 |
| **Working status of respondent** | |  |
| Not Working® |  |  |
| Working | 1.14 | 0.88 |
| **Respondent owns a bank account** | | |
| No® |  |  |
| Yes | 2.79 | 0.36 |
| **Respondent owns a mobile phone** | | |
| No® |  |  |
| Yes | 1.44 | 0.69 |
| **Community level variables** | |  |
| **Proportion of women with secondary level of education in PSU** | | |
| 0-25%® |  |  |
| 26-50% | 1.55 | 0.65 |
| >50% | 1.92 | 0.52 |
| **Proportion of poor women in PSU** | | |
| 0-25%® |  |  |
| 26-50% | 1.53 | 0.65 |
| >50% | 2.72 | 0.37 |
| Mean VIF | 1.81 |  |

Notes: ®= reference category, VIF= Variance inflation factors
